# Supplementary figures and images for: Clathrin-independent endocytic retrieval of SV proteins mediated by the clathrin adaptor AP-2 at mammalian central synapses
Source: eLife. 2022 Jan 11;11:e71198. doi: 10.7554/eLife.71198 (PMC8752090; doi:10.7554/eLife.71198)

Figure 2\_figure Supplement 1C- Blot

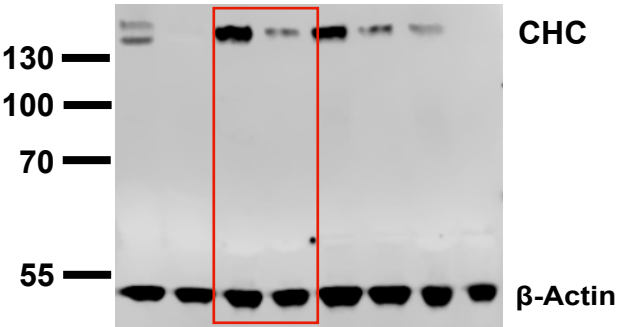

Supplement: Figure 2—figure supplement 1—source data 2. [file elife-71198-fig2-figsupp1-data2.pdf]

## Figure 6 Blots

Figure 6B

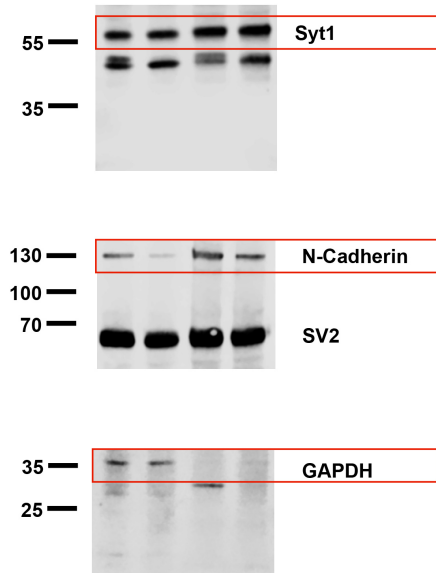

Figure 6D

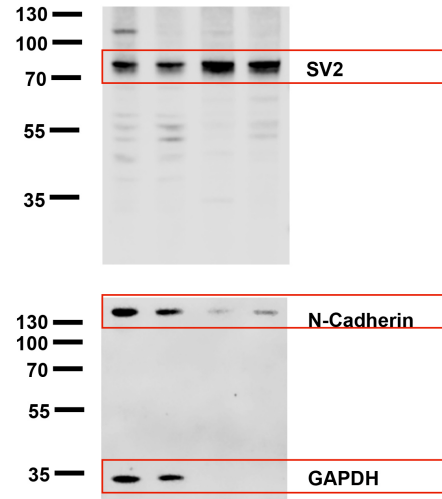

Figure 6F

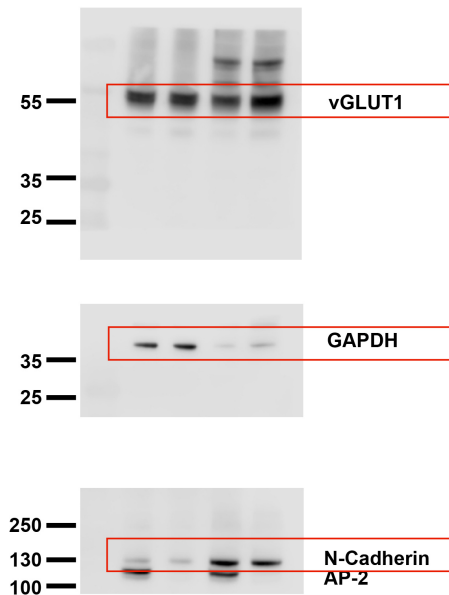

Figure 6H

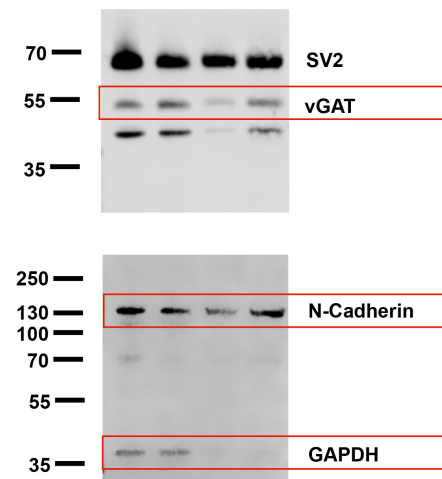

Supplement: Figure 6—source data 2. [file elife-71198-fig6-data2.pdf]

Figure 8B and C, and figure supplement 6A - Blots

### AP-1

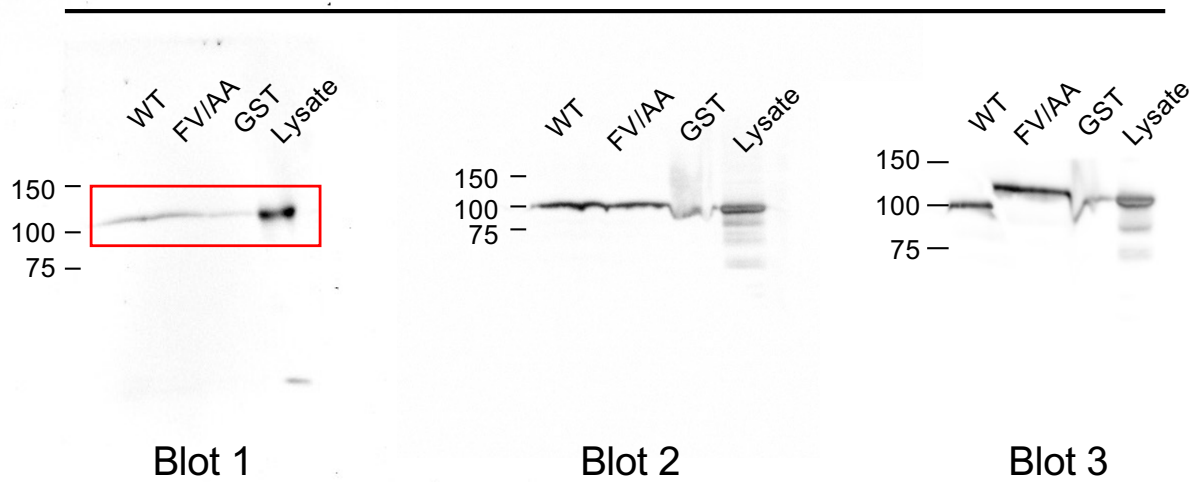

### AP-2

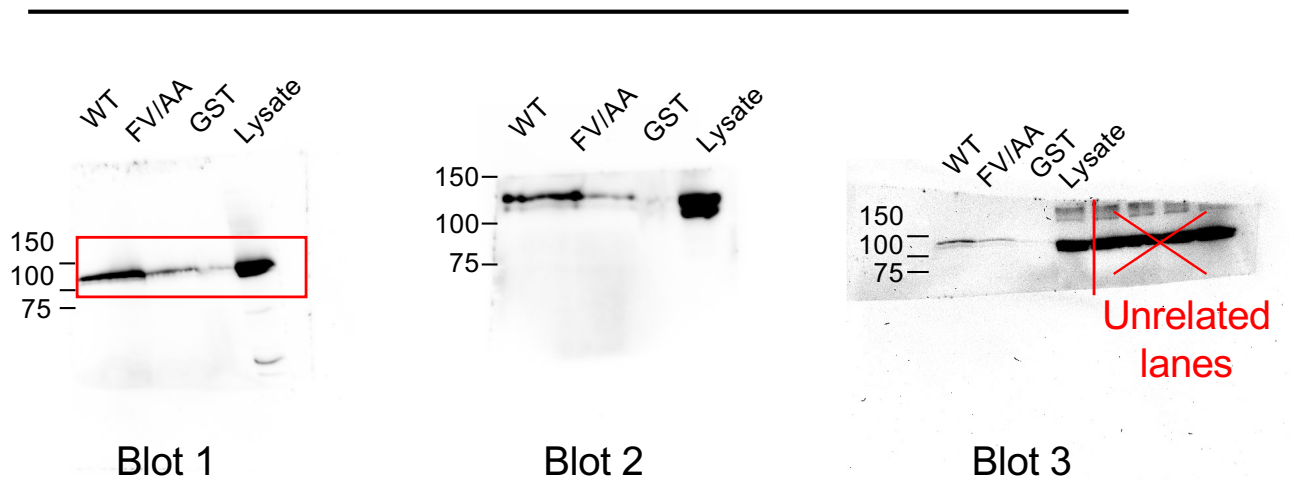

### AP-3

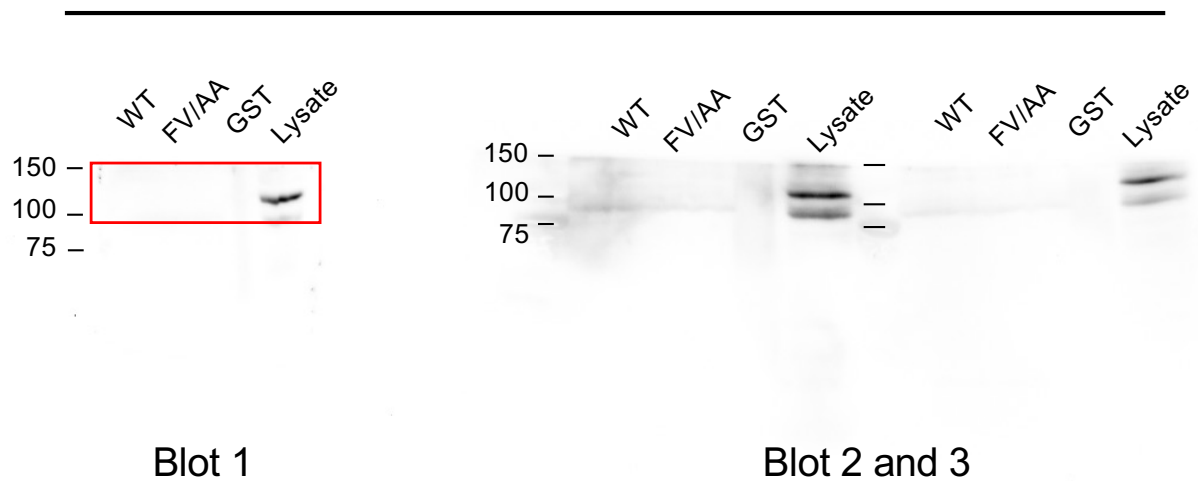

Supplement: Figure 8—source data 2. [file elife-71198-fig8-data2.pdf]
